# Induced pluripotent stem cells-derived microvesicles accelerate deep second-degree burn wound healing in mice through miR-16-5p-mediated promotion of keratinocytes migration

**DOI:** 10.7150/thno.46639

**Published:** 2020-08-08

**Authors:** Yuan Yan, Ruijun Wu, Yunyao Bo, Min Zhang, Yinghua Chen, Xueer Wang, Mianbo Huang, Baiting Liu, Lin Zhang

**Affiliations:** 1 Department of Histology and Embryology, School of Basic Medical Science, Southern Medical University, Guangzhou 510515, China.; 2 Guangdong Provincial Key Laboratory of Construction and Detection in Tissue Engineering, Guangzhou 510515, China.

**Keywords:** iPSCs, microvesicles, burn wound healing, miR-16-5p, migration

## Abstract

**Background:** Induced pluripotent stem cells (iPSCs) have emerged as a promising treatment paradigm for skin wounds. Extracellular vesicles are now recognized as key mediators of beneficial stem cells paracrine effects. In this study, we investigated the effect of iPSCs-derived microvesicles (iPSCs-MVs) on deep second-degree burn wound healing and explored the underlying mechanism.

**Methods:** iPSCs-MVs were isolated and purified from conditioned medium of iPSCs and confirmed by electron micrograph and size distribution. In deep second-degree burn model, iPSCs-MVs were injected subcutaneously around wound sites and the efficacy was assessed by measuring wound closure areas, histological examination and immunohistochemistry staining. *In vitro*, CCK-8, EdU staining and scratch assays were used to assess the effects of iPSCs-MVs on proliferation and migration of keratinocytes. Next, we explored the underlying mechanisms by high-throughput microRNA sequencing. The roles of the miR-16-5p in regulation of keratinocytes function induced by iPSCs-MVs were assessed. Moreover, the target gene which mediated the biological effects of miR-16-5p in keratinocytes was also been detected. Finally, we examined the effect of local miR-16-5p treatment on deep second degree-burns wound healing in mice.

**Results:** The local transplantation of iPSCs-MVs into the burn wound bed resulted in accelerated wound closure including the increased re-epithelialization. *In vitro*, iPSCs-MVs could promote the migration of keratinocytes. We also found that miR-16-5p is a critical factor in iPSCs-MVs-induced promotion of keratinocytes migration *in vitro* through activating p38/MARK pathway by targeting Desmoglein 3 (Dsg3). Finally, we confirmed that local miR-16-5p treatment could boost re-epithelialization during burn wound healing.

**Conclusion:** Therefore, our results indicate that iPSCs-MVs-derived miR-16-5p may be a novel therapeutic approach for deep second-degree burn wound healing.

## Introduction

Rapid and efficient closure of wounds is essential for maintaining skin integrity and prevent systemic invasion by infectious agents. Normal wound healing is one of the most complex biological processes, which requires the accurate cooperation of many types of cells and the precise coordination of various biological and molecular events 1-3. Although various therapeutic attempts have been made to promote wound healing, optimal treatment strategies are still being developed.

Stem cell-based therapy has opened a new door for tissue repair and has been extensively studied in the field of regenerative medicine. Stem cells from numerous sources are currently being tested in preclinical and clinical trials for their ability to promote wound healing and tissue regeneration 4-6. Interestingly, many investigators have demonstrated that stem cell transplantation therapy promotes wound healing mainly through the paracrine mechanism 7-10 and extracellular vesicles play a major role in this mechanism 11. Increasing evidence has suggested that the application of extracellular vesicles derived from Mesenchymal stem cell (MSCs) and other cell types for acceleration of the wound healing process have shown promising results 12-15.

Extracellular vesicles are heterogeneous bilayer membrane structures comprising exosomes (30-150 nm) and microvesicles (100-1000 nm). They are perceived as mediators for intercellular communication, allowing biologically active molecules, such as microRNAs (miRNAs), messenger RNAs (mRNAs), and proteins, to be exchanged to targeted recipient cells and to reprogram cell behaviors 16-18. Among these molecules, miRNAs have attracted most attention, due to their important modulators of gene expression and physiological changes they cause in recipient cells 19, 20. Many studies have shown that MSCs-derived extracellular vesicles play a role in regulating inflammatory response, promoting the formation of vascularized granulation matrix, and increasing the proliferation and migration of skin cells through specific microRNAs (miR-181c, miR-21, miR-125b, miR-145, miR-146a, miR-23a, etc.) 21-23. Therefore, a better understanding of the miRNA expression profiles and putative specific miRNA functions within EVs will facilitate further development of stem cells-mediated extracellular vesicles therapy.

Induced pluripotent stem cells (iPSCs) show unlimited growth capacity, are not associated with ethical issues, are superior to traditional epidermal stem cells (ESCs) and MSCs, and can serve as an inexhaustible source for stem cell transplantation therapy 24-26. Kobayashi and colleagues had demonstrated that the exosomes derived from the iPSCs has beneficial effects on skin wound healing 15. However, little is known about the content of iPSCs-derived extracellular vesicles and their relationship to specific functions of EVs.

Therefore, in the present study, we isolated extracellular vesicles from condition medium of iPSCs and confirmed as microvesicles. Then we verified the therapeutic effects of iPSCs-derived microvesicles (iPSCs-MVs) in deep second-degree burn wound healing. The results showed that the iPSCs-MVs could promote the keratinocytes migration *in vivo* and *in vitro*. Furthermore, through high-throughput sequencing, we analyzed miRNA profiles in iPSCs-MVs. We found that miR-16-5p was enriched in iPSC-EVs and this miRNA was a key mediator in the iPSCs-MVs-induced regulation of keratinocytes migration by targeting Dsg3. The findings show a potential role of iPSC-EVs and miR-16-5p in cutaneous wound healing.

## Methods

### Cell culture and transfection

The mouse iPS cell line OSKM-1 was provided by Stem Cell Bank, Chinese Academy of Sciences. iPSCs were directly adapted to serum free, feeder-free expansion medium by dissociation cells with Accutase (Millipore, USA) and passaging them into 0.1% gelatin coated plate containing ESCRO Complete Plus Medium (Millipore, USA). Replace with fresh ESGRO Complete Plus Media every other day. HaCaT cells were cultured in Dulbecco's modified Eagle's medium (DMEM, HyClone, USA) supplemented with 1% non-essential amino acid and 10% fetal bovine serum (Gibico, USA) under 5% CO_2_ at 37 °C. Transfection of miR-16-5p mimics/inhibitor (RiBoBio, China), scrambled miRNA (negative control [NC]), Dsg3 siRNA, or pcDNA3.1-Dsg3 plasmid was carried out by using Lipofectamine 2000 (Invitrogen, USA) according to the manufacturer's instructions.

### Isolation and labeling characterization of iPSCs-MVs

iPSCs were cultured in ESCRO Complete Plus Medium for 48 h. The conditioned medium was collected and microvesicles were isolated by using ExoEasy Maxi Kit (Qiagen, Germany) according to the manufacturer's instructions. The purified microvesicles fraction was re-suspended in PBS and stored at -80 °C for further experimental needs. 1 μL exosomes were used to quantitate their concentration by BCA protein assay kit (Thermo Scientific, USA), as suggested by the manufacturer. Morphology of iPSCs-MVs was visualized by transmission electron microscopy (TEM). First, iPSCs-MVs were placed on carbon-coated grid for 10 min and fixed with 3% paraformaldehyde. After negative staining with 2% uranylacetate for 10 min, iPSCs-MVs was observed with the JEM-1200 electron microscope (JEOL, Japan), operated at 100 KV. Size distribution and concentration of iPSCs-MVs were analyzed by nanoparticle tracking analysis (NTA) using Nanosight LM 10 (Malvern Panalytical, UK). The mode, mean size, and concentration of iPSCs-MVs were determined by NTA 3.2 Dev Build 3.2.16 software.

### Deep second-degree burns in mice and treatments

C57BL/6 mice aged 6 to 8 weeks were purchased from the Laboratory Animal Centre of Southern Medical University. A deep second-degree burn was made as previously described 27. Briefly, after hair removal from the bilateral sites of the abdomens of mice, the opening at one end of a cylindrical plastic tube having a diameter of 1 cm was attached to the skin of the abdomen of mice. The boiling water was then poured into the tube at a height of 2 cm and taken out after 15 s. The mice received intradermal injection of PBS, iPSCs-MVs, miR-16-5p agomir or agomir NC immediately after burn. The wounds were recorded with a digital camera.

### Histological assessment

The skin samples were fixed in 4% paraformaldehyde for 48 h, embedded in paraffin, and then cut into 5 μm thick tissue sections. The sections were used for hematoxylin and eosin (H&E) staining according to the manufacturer's manual. Re-epithelialization was calculated according to the following formula: [distance of the minor axis covered by the epithelium]/ [distance of the minor axis between the edges of the original wound] ×100.

### *In vivo* tracking experiment

Purified microvesicles were labeled with the PKH67 Green Fluorescent Cell Linker Kit (Sigma-Aldrich, USA) according to the manufacture's protocol. The burn wounds on mice were injected with PBS or PKH67-labeled iPSCs-MVs immediately after burn. Fluorescence images were taken at days 1, 3 and 5 by the *in vivo* imaging system (Bruker, USA). The images were analyzed using Bruker MI SE 7.2 software.

### Immunohistochemical assay

For immunohistochemical staining, the tissue sections were dewaxed, and then incubated with 3% H_2_O_2_ for 20 min, and the antigen was restored by heating in citrate buffer (pH 6.0). The sections were then blocked with goat serum for 30 min and incubated overnight at 4 °C with primary antibodies against K6, α-SMA, CD31, CD68 or Dsg3 (Abcam, UK). On the second day, the sections were treated with biotinylated secondary antibodies (Zhong Shan Golden Bridge Biotechnology, China) for 30 min at room temperature. Peroxidase activity was detected by diaminobenzidine (DAB). Finally, sections were counterstained with hematoxylin.

### Internalization of iPSCs-MVs by HaCaT cells

PKH67-labeled microvesicles were co-cultured with HaCaT cells in FBS-free medium for 10 h. The internalization of microvesicles by HaCaT cells were counterstain with 4,6-diamidino-2-phenylindole (DAPI, 500 ng/mL), and observed by the fluorescence microscope (Leica, Germany).

### CCK-8 assay

Cell were plated at a density of 2×10^3^ cells per well on 96-well plates in 100 μL of culture medium. At the indicated time, CCK-8 (10 µL, Beyotime, China) was added to each well and incubated for an additional 4 h. The OD was measured with a DTX-880 Multimode Detector (Beckman Coulter, USA) at the wave length of 450 nm.

### EdU staining

For EdU staining, EdU was added to the culture medium for 4 h in order to incorporate into replicating cells' DNA. Cells were washed and then fixed with 4% paraformaldehyde for 15 min. 0.2% Triton X-100 was used to permeabilize the nuclear membrane. Ultimately, cells were stained by Cell-Light^TM^ Apollo488 Stain Kit (RiBoBio, China) according to the manufacturer's instructions. Cells were detected with a fluorescence microscope (Leica, Germany). For cell number counting, at least 200 cells or 10 images were quantified in each well to get accurate numbers for each group.

### Scratch wound healing assay

Cells were cultured in 24-well plates overnight. Linear scratch wounds were created by 200 μL sterile pipette tip when each well was filled with cells. The drifting cells were washed away and removed by PBS. After cells were replenished with fresh medium, the wound healing status was observed and photographed at 24 h post-wounding. The wound-healing rate was quantitatively evaluated using the Image J software.

### miRNA microarray analysis

Total RNA was isolated from iPSCs-MVs using the miRNeasy Micro Kit (Qiagen, USA). cDNA libraries were generated using the NEBNext Multiplex Small RNA Library Prep Set for Illumina (New England Biolabs). The amplified libraries were size selected using a 5% polyacrylamide gel and purified using the QIAQuick PCR Purification Kit (Qiagen, Germany), according to the manufacture's protocol. Purified libraries were normalized and pooled to create a double stranded cDNA library ready for sequencing. The samples were sequenced using the Illumina HiSeq^TM^ 2500 DNA sequence analyzer. Adapter sequences were removed and low quality reads were trimmed from raw sequencing reads using Cutadapt (v. 1.11). The resulting reads were mapped to the primary assembly of the mouse genome, Rfam11.0, and miRBase (version: 22.0) using BWA. Reads were normalized to reads per million reads (RPM).

### Western blot analysis

The total proteins were lysed in ice-cold Radio-Immunoprecipitation Assay (RIPA, Sigma) lysis buffer and separated by electrophoresis on sodium dodecyl sulfate-polyacrylamide gel electrophoresis (SDS-PAGE) gel and transferred to a polyvinylidene difluoride membrane (Millipore, USA). After blocking with 5% fat-free milk, the proteins were incubated with antibodies for Annexin A1, TSG101, ARF6, β-actin, p38, p-p38(Abcam, UK) overnight at 4 ℃. Subsequently, the membranes were incubated with horseradish peroxidase conjugated secondary antibodies (Thermo Fisher Scientific, USA). The bands were visualized using the ECL detection system (Millipore, USA). Quantity One software (Bio-Rad) was used to detect the band intensity.

### Luciferase reporter assay

The 3'-UTR or the mutated 3'-UTR sequence of Dsg3 was amplified by PCR from human genomic DNA and cloned into the psiCHECK-2 vector. All plasmids were confirmed by DNA sequencing. For reporter assays, HEK-293 T cells were seeded in 24-well plates and co-transfected with the constructed luciferase report vector and miR-16-5p mimics or negative control oligoribonucleotides (mimics NC) using Lipofectamine 2000 (Invitrogen, USA) following the manufacturer's instructions. After 48 h, dual luciferase reporter assays (Promega, USA) were used to detect Firefly and Renilla luciferase activities in cell lysates according to the manufacturer's instructions.

### Quantitative real-time polymerase chain reaction (qRT-PCR)

Total RNA extraction from HaCaT cells was performed with Trizol™ Reagent (Invitrogen), according to the manufacturer's instructions. cDNA was synthesized from 1 μg RNA using a HiScript II Q RT SuperMix for qPCR (Vazyme, China). Then, the qRT-PCR analysis was performed with AceQ Universal SYBR qPCR Master Mix (Vazyme, China) on an iCycler System (Bio-Rad, USA). The Ct values were normalized for the housekeeping gene GAPDH. The sequences of the primers were as follows: Dsg3 upstream, 5'- CACCTACCGAATCTCTGGAGT -3', and downstream, 5'-GGGCATTTAGAGCCCGACA-3'; GAPDH upstream, 5'-CTGGGCTACACTGA-GCACC-3', and downstream, 5'- AAGTGGTCGTTGAGGGCAATG -3'. For miRNA analysis, cDNA for miRNA was synthesized using the miDETECT A Track^TM^ miRNA qRT-PCR Starter Kit (Ribobio, China) as described by the manufacture's protocol. The U6 RNA level was used as an internal control for data normalization. The qRT-PCR reaction was performed using miDETECT A Track^TM^ miRNA qPCR Kit (Ribobio, China) with the miDETECT A Track^TM^ miR-16-5p Forward Primer and miDETECT A Track^TM^ Uni-Reverse Primer (Ribobio, China).

### Statistical analysis

All data were reported as mean ± standard deviation (SD) of at least three independent experiments (n ≥ 3). Statistical analysis was performed by independent samples t-test for comparison between two groups or one-way ANOVA among the groups. Values of *p* < 0.05 were considered to be statistically significant.

## Results

### Characterization of iPSCs-MVs

**Figure 1:**
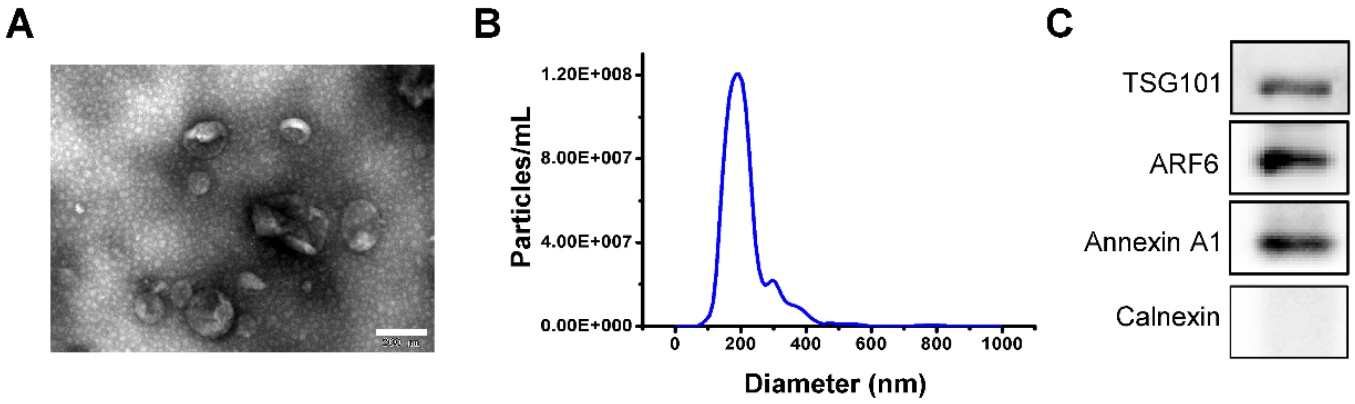
** Characterization of iPSCs-MVs.** (**A**) The transmission electron microscope (TEM) image of iPSCs-MVs. Scale bar = 200 nm (**B**) Nanoparticle tracking analysis (NTA) result of iPSCs-MVs. Mean diameter of iPSCs-MVs was 214.6 ± 8.3 nm. (**C**) Microvesicles marker proteins TSG101, ARF6 and Annexin A1 were identified by western blot. Calnexin was used as an internal reference.

Extracellular vesicles secreted from iPSCs were isolated and then characterized by morphology and size. Transmission electron microscopy (TEM) revealed that iPSCs-derived extracellular vesicles were primarily circular and double membrane wrapped in shape (**Figure 1A**). We utilized nanoparticle tracking analysis (NTA) to evaluate extracellular vesicles numbers and their size profiles. The NTA results showed that the size distribution of iPSCs-derived extracellular vesicles had a major peak at 186 nm and the mean diameter was 214.6 nm (**Figure 1B**). The protein of the iPSCs-MVs was positive for the microvesicles markers Annexin A1, TSG101 and ARF6 28, while negative for the endoplasmic reticulum protein, calnexin (**Figure 1C**). All these data indicate that most of the extracellular vesicles used in this study were microvesicles.

### Retention of iPSCs-MVs in skin tissues

**Figure 2:**
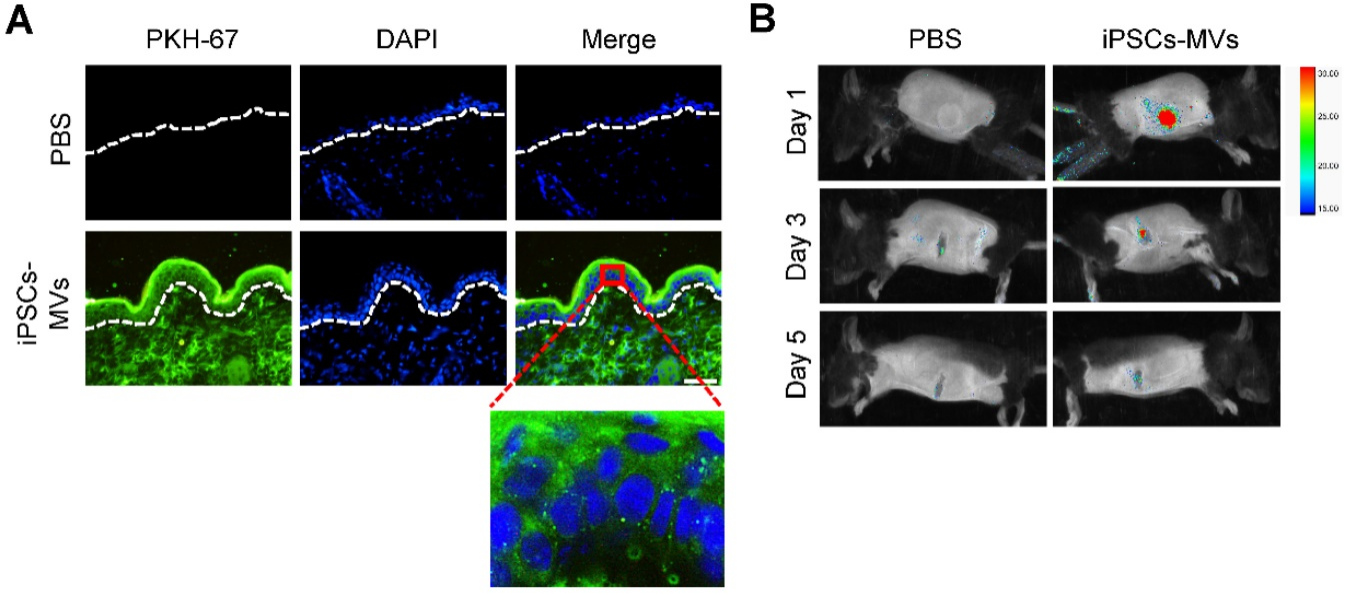
** Retention of iPSCs-MVs in skin tissues.** (**A**) Representative images of iPSCs-MVs incorporation in skin tissues on days 1 after wounding. Scale bar = 50 µm. (**B**) Representative fluorescence imaging of mice wounds treated with PKH67-labeled iPSCs-MVs or PBS on days 1, 3, and 5 after wounding.

Before the start of follow experiments, we tested the retention of the administered iPSCs-MVs in skin tissues. As detected by fluorescence microscopy, plenty of PKH67-labeled iPSCs-MVs were observed in the cytoplasm of epidermal cells at 24 h after injection (**Figure 2A**). The results indicated that the transplanted iPSCs-MVs were uptake by the resident epidermal cells. In addition, the *in vivo* tracking experiments showed that signals could still be acquired on day 3 after injection, while no signals were detected on day 5 (**Figure 2B**), suggesting that the retention of transplanted iPSCs-MVs was time-dependent decrease.

### iPSCs-MVs accelerate deep second-degree burn wound healing

**Figure 3:**
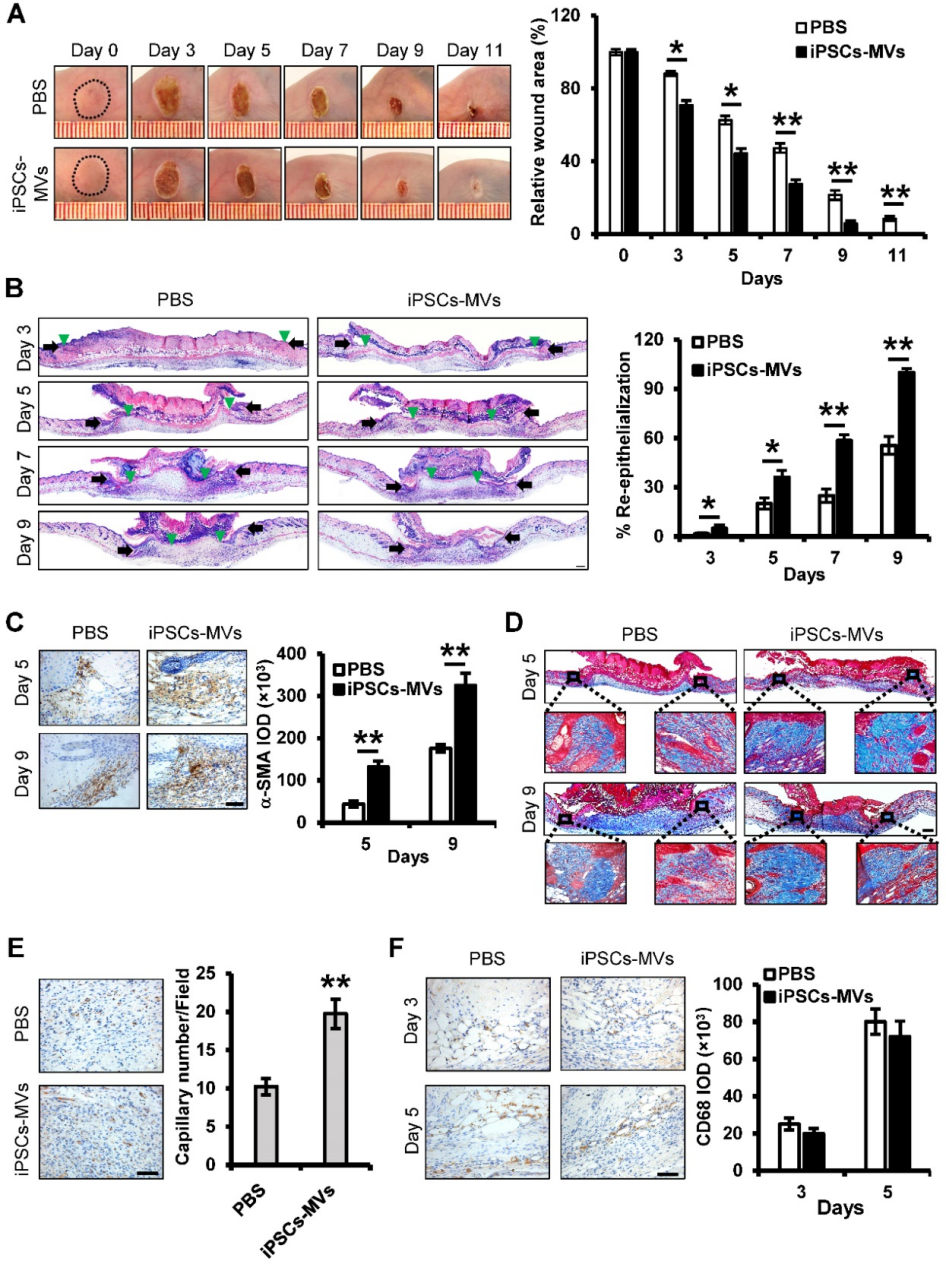
** iPSCs-MVs accelerate deep second-degree burn wound healing and promote keratinocytes migration *in vivo*.** (**A**) Representative macroscopic images of wounds treated with PBS or iPSCs-MVs on days 0, 3, 5, 7, 9 and 11 after wounding (left panel). Quantitative analysis of wound area per group, expressed as the percentage of the initial wound size at day 0 (right panel). n = 6 mice per group. (**B**) Representative photomicrographs of H&E-stained wounds per group on days 0, 3, 5, 7 and 9 after wounding. Black arrows represent the dermal border; green arrows represent the epidermal margin (left panel). Scale bar = 200 µm. Quantitative profiles of the re-epithelialization ration of wounds (right panel). The re-epithelialization was calculated as described in Materials and Methods. (**C**) Representative photomicrographs of α-SMA immunostaining of wounds per group on days 5 and 9 after wounding (left panel). Scale bar = 50 µm. The areas stained with α-SMA were determined by planimetric image analysis using Image Pro Plus 6.0 software (right panel). (**D**) Representative photomicrographs of Masson's trichrome-stained wounds per group on days 5 and 9 after wounding. (**E**) Representative photomicrographs of CD31 immunostaining of wounds per group on days 11 after wounding (left panel). Scale bar = 50 µm. The numbers of stained capillaries were counted (right panel). Statistics regarding the number of stained capillaries were obtained using five randomly selected fields of view for each group. (**F**) Representative photomicrographs of CD68 immunostaining of wounds per group on days 3 and 5 after wounding (left panel). Scale bar = 50 µm. The areas stained with CD68 were determined by planimetric image analysis using Image Pro Plus 6.0 software (right panel). All values are expressed as mean ± SD from three independently repeats, *P < 0.05, **P < 0.01 compared with control.

To evaluate the effect of iPSCs-MVs on burn wound healing, deep second-degree burn were created on the abdominal skin, following by local injection of iPSCs-MVs or equal amounts of PBS as control. Macroscopic evaluation showed that iPSCs-MVs significantly promoted the rates of wound closure compared with PBS on days 3 to 11 after treatment (**Figure 3A**). Then, tissues of wound area were biopsied for histological examination and immunohistochemistry staining. H&E staining revealed that re-epithelialization was significantly enhanced by iPSCs-MVs (**Figure 3B**). We further examined the impact of iPSCs-MV on other key biological processes. Immunofluorescence staining for a-SMA and Masson's staining showed more myofibroblasts and collagen deposition in the wounds treated with iPSCs-MVs compared with the PBS group (**Figure 3C, D**). In addition, immunofluorescence staining for CD31 showed that the number of vessels was increased in iPSCs-MVs group (**Figure 3E**). However, there was no significant difference in the amount of macrophages identified by CD68 immunostaining between the iPSCs-MVs group and the PBS group (**Figure 3F**). Collectively, these results suggest that local iPSCs-MVs treatment could accelerate the process of wound healing through increased re-epithelialization, fibrogenesis and angiogenesis.

### iPSCs-MVs promote keratinocytes migration *in vivo*

**Figure 4:**
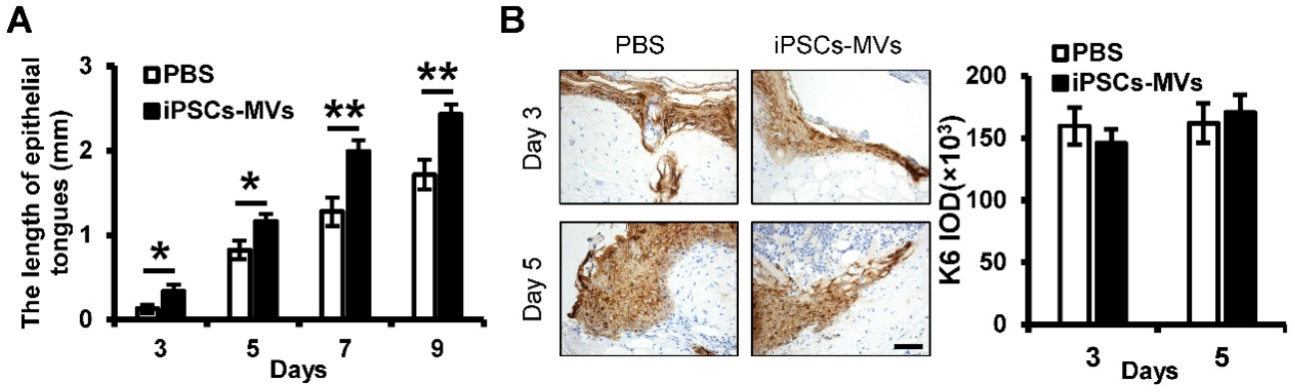
** iPSCs-MVs promote keratinocytes migration *in vivo*.** (**A**) Quantitative profiles of the length of epithelial tongues of wounds treated with PBS or iPSCs-MVs on days 3, 5, 7 and 9. (**B**) Representative photomicrographs of K6 immunostaining of wounds treated with PBS or iPSCs-MVs on days 3 and 5 after wounding (left panel). Scale bar = 50 µm. The areas stained with K6 were determined by planimetric image analysis using Image Pro Plus 6.0 software (right panel). All values are expressed as mean ± SD from three independently repeats. *P < 0.05, **P < 0.01 compared with control.

An essential feature of a healed wound is re-epithelialization, which relies on two basic functions of keratinocytes: proliferation and migration 29. To investigate the mechanism by which iPSCs-MVs accelerated re-epithelialization, we observed the effect of iPSCs-MVs on proliferation and migration of keratinocytes *in vivo*. As shown in **Figure 4A**, there was a significant increase in the length of epithelial tongues on days 3, 5, 7 and 9 in the iPSCs-MVs group compared to the PBS group. However, K6 staining of the wound site showed no significant difference in proliferating keratinocytes in wound sites between the iPSCs-MVs group and PBS group on days 3 and 5 after the injury (**Figure 4B**). All above results suggest that iPSCs-MVs could increase re-epithelization mainly *via* the accelerated keratinocytes migration, but not proliferation.

### iPSCs-MVs promote keratinocytes migration *in vitro*

**Figure 5:**
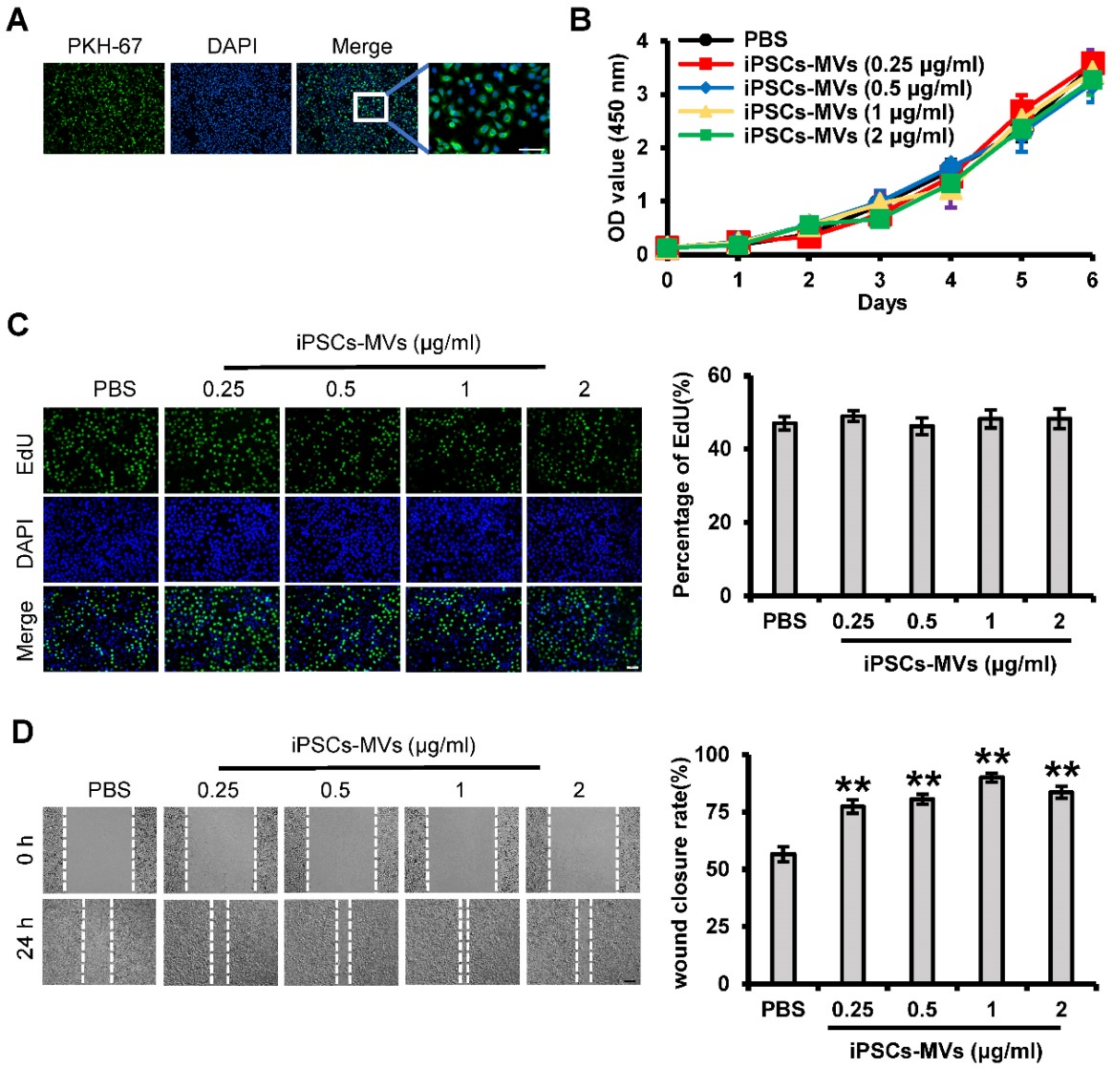
** iPSCs-MVs are taken up by HaCaT cells and promote keratinocytes migration *in vitro*.** (**A**) Representative fluorescence imaging of HaCaT cells incubated with either PBS or PKH67-labeled iPSCs-MVs for 10 h. Scale bar = 100 µm. (**B**) The proliferative ability of HaCaT cells treated with PBS or different concentrations of iPSCs-MVs (0.25, 0.5, 1, or 2 µg/mL) was measured by CCK-8 assay. (**C**) Representative fluorescence imaging of EdU staining of HaCaT cells treated with PBS or different concentrations of iPSCs-MVs (0.25, 0.5, 1, or 2 µg/mL) for 24 h (left panel). Scale bar = 100 µm. The proliferation rates were quantified by percentage of EdU-positive HaCaT cells (right panel). (**D**) Scratch wound healing assays were performed to assess the migration rate of HaCaT cells treated with PBS or different concentrations of iPSCs-MVs (0.25, 0.5, 1, or 2 µg/mL) for 24 h. Photographs were taken at 24 h after scratch injury (left panel). Scale bar = 200 µm. The healing rates were quantified by measuring the area of the injured region (right panel). All values are expressed as mean ± SD from three independently repeats. **P < 0.01 compared with control.

We next examined whether the iPSCs-MVs had effects on proliferation or migration of keratinocytes *in vitro*. First, we also verified the integration ability of iPSCs-MVs by PKH67 assay, using a membrane labeling dye (PKH67) that integrates specifically into the membrane bilayer structure during fusion. After incubating HaCaT cells, a well‐established immortalized human keratinocyte cell lines, with iPSCs-MVs for 10 h, the PKH67-labeled MVs were observed in the cytoplasm of HaCaT cells *via* fluorescence microscopy (**Figure 5A**), indicating that iPSCs-MVs can be internalized by keratinocytes.

CCK-8 and EdU assays were applied to determine the effect of iPSCs-MVs on the proliferation of HaCaT cells. Consistent with in-vivo results, iPSCs-MVs also had no effect on cell proliferation *in vitro* (**Figure 5B-C**). We further studied iPSCs-MVs in their ability to induce keratinocytes migration during the wound-healing process by using the scratch assay, an *in vitro* procedure used to study cell migration. The migration rate of HaCaT cells after iPSCs-MVs treatment was found to be significantly increased at all doses as compared to PBS. Further, a dose-response was noted with the 1 μg/mL dose demonstrating the greatest migration rate (**Figure 5D**). Taken together, these results suggest that iPSCs-MVs can promote keratinocytes migration, but have no effect on keratinocytes proliferation *in vitro*.

### miR-16-5p is abundant in iPSCs-MVs and plays a key role in enhanced keratinocytes migration induced by iPSCs-MVs

**Figure 6:**
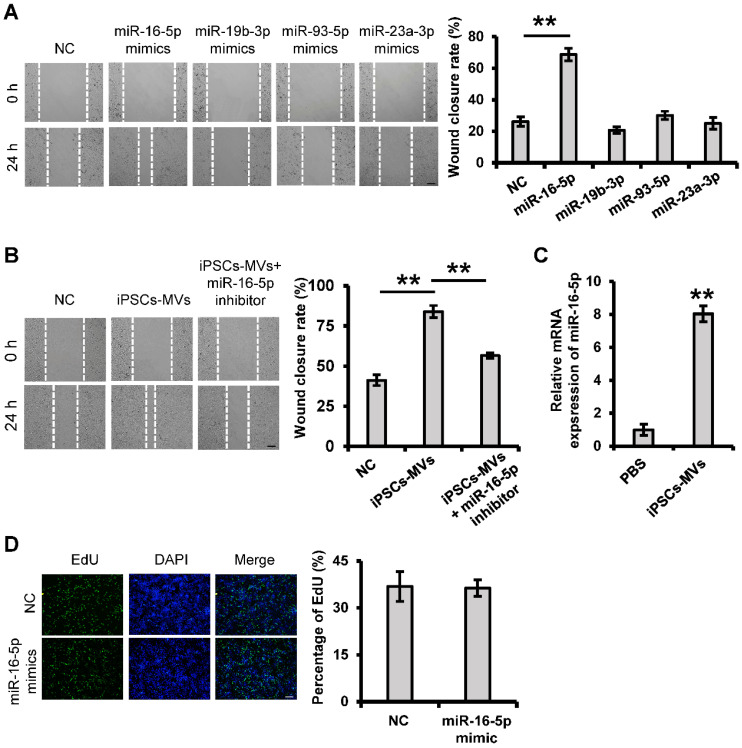
** iPSCs-MVs-derived miR-16-5p promotes keratinocytes migration *in vitro*.** (**A**) Scratch wound healing assays were performed to detect the migration of HaCaT cells transfected with miR-16-5p mimics, miR-19b-3p mimics, miR-93-5p mimics, miR-23a-3p mimics or miRNA mimics negative control (mimics NC) for 48 h. Photographs were taken at 24 h after scratch injury (left panel). Scale bar = 200 µm. The healing rates were quantified by measuring the area of the injured region (right panel). (**B**) Scratch wound healing assays were performed to assess the migration rate of keratinocytes transfected with miR-16-5p inhibitor for 48 h in the absence or presence of iPSCs-MVs. Photographs were taken at 24 h after scratch injury (left panel). Scale bar = 200 µm. The healing rates were quantified by measuring the area of the injured region (right panel). (**C**) The miR-16-5p expression was detected in HaCaT cells after incubation with iPSCs-MVs for 24 h by qRT-PCR. (**D**) Representative fluorescence imaging of EdU staining of HaCaT cells treated with mimics NC or miR-16-5p mimics for 48 h (left panel). Scale bar = 200 µm. The proliferation rates were quantified by percentage of EdU-positive HaCaT cells (right panel). All values are expressed as mean ± SD from three independently repeats, **P < 0.01 compared with control.

It has previously been shown that the main type of functional RNA component in extracellular vesicles is microRNA, which can be efficiently transmitted to other cells and achieve diverse functions through extracellular vesicles integration. To investigate whether miRNA within iPSCs-MVs is important in their pro-migration effects, we took the unbiased approach of sequencing the miRNA. Among the most abundant 10 miRNAs in the iPSCs-MVs (**Table S1**), miR-16-5p, miR-93-5p, miR-19b-3p and miR-23a-3p had been known to have promotion on cell migration 29-33. Using the scratch assay, we found that overexpression of miR-16-5p, which was the most highly expressed miRNA in iPSCs-MVs, significantly accelerated the healing rate of HaCaT cells 24 h after scratching (**Figure 6A**). To further verify the role of miR-16-5p in the enhanced keratinocytes migration induced by iPSCs-MVs, HaCaT cells stimulated with iPSCs-MVs were additionally treated with a specific inhibitor targeting miR-16-5p. As illustrated in **Figure 6B**, the pro-migratory effect of iPSCs-MVs was attenuated in the iPSCs-MVs plus miR-16-5p inhibitor group. In addition, we found the miR-16-5p expression levels was indeed significantly increased in the HaCaT cells after treating them with iPSCs-MVs for 48 h compared with control group (**Figure 6C**), supporting that iPSCs-MVs could deliver miR-16-5p to the recipient cells. Interesting, there was no significant difference in the percentage of EdU-positive proliferating keratinocytes between the miR-16-5p mimics treated group and mimics NC treated group (**Figure 6D**), suggesting that miR-16-5p has no effect on keratinocytes proliferation. Taken together, all of these results reveal that miR-16-5p plays a key role in enhanced keratinocytes migration induced by iPSCs-MVs.

### miR-16-5p promotes keratinocytes migration through activating p38/MARK pathway by targeting Dsg3

**Figure 7:**
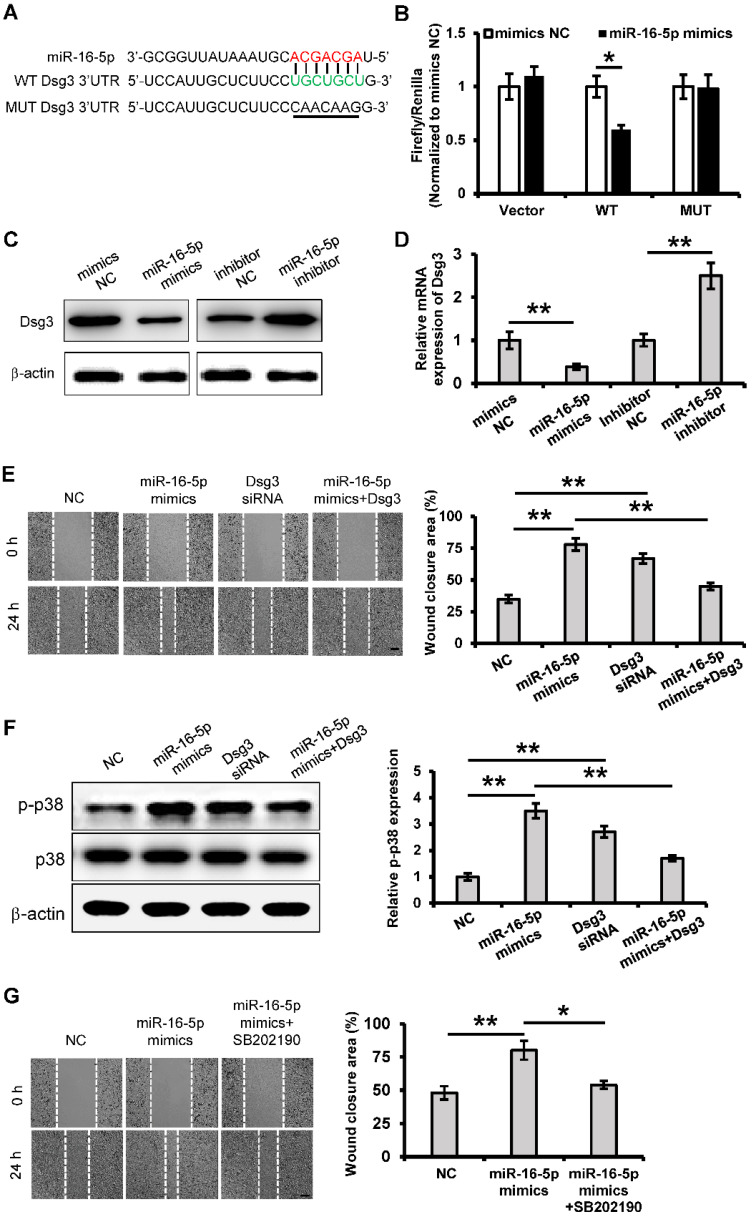
** miR-16-5p promotes keratinocytes migration by targeting Dsg3. (A)** Predicted miR-16-5p target sequences in Dsg3 3'-UTR in human and positions of mutated nucleotides in the 3'UTR of DSG3. (**B**) Luciferase reporter assay determined luciferase activity in 293T cells co-transfected with miR-16-5p mimics and psiCHECK-Dsg3-wt-3'UTR (WT) or psiCHECK-Dsg3-mut-3'UTR (MUT). (**C**) Western blot analysis of Dsg3 expression in HaCaT cells transfecting mimics negative control (mimics NC), miR-16-5p mimics or miR-16-5p inhibitor, β-actin was used as the loading control. (**D**) qRT-PCR analysis of Dsg3 expression in HaCaT cells transfecting miR-16-5p mimics or miR-16-5p inhibitor. (**E**) Scratch wound healing assays were performed to assess the migration rate of HaCaT cells transfected with Dsg3 siRNA, miR-16-5p mimics, or miR-16-5p mimics plus pcDNA3.1-Dsg3 for 48 h. Photographs were taken at 24 h after scratch injury (left panel). Scale bar = 200 µm. The healing rates were quantified by measuring the area of the injured region (right panel). (**F**) Western blot analysis of p-p38 and p38 expression in HaCaT cells transfected with Dsg3 siRNA, miR-16-5p mimics, or miR-16-5p mimics plus pcDNA-Dsg3 for 48 h. β-actin was used as the loading control. (**G**) Scratch wound healing assays were performed to assess the migration rate of in HaCaT cells transfected with miR-16-5p mimics in the absence or presence of p38MAPK-specific inhibitor SB202190. Photographs were taken at 24 h after scratch injury (left panel). Scale bar = 200 µm. The healing rates were quantified by measuring the area of the injured region (right panel). All values are expressed as mean ± SD from three independently repeats, *P < 0.05, **P < 0.01.

To determine the regulatory mechanism of the role of miR-16-5p in keratinocytes migration, we tried to predict the target gene of miR-16-5p by using bioinformatics analysis (Targetscan, miRMap and miRanda). In this study, we focused on one of them, Desmoglein 3 (Dsg3), which is a desmosomal adhesion protein that has been shown to regulate keratinocytes migration and wound healing 34. According to the Target Scan analysis, Dsg3 has a miR-16-5p binding site in its 3'-UTR (**Figure 7A**). To determine whether miR-16-5p binds directly to the 3'UTR of Dsg3 to affect its expression, the wild Dsg3 3'UTR (WT) and the mutated (MUT) Dsg3 3'UTR were reconstituted into the psiCHECK-2 vector. When introduced into 293T cells, the WT Dsg3 3'UTR reporter showed a significant reduction in luciferase activity in the miR-16-5p mimics-transfected cells (**Figure 7B**). However, the MUT Dsg3 3'UTR did not change significantly (**Figure 7B**). To further validate whether miR-16-5p directly targets Dsg3, we evaluated the role of miR-16-5p in the endogenous Dsg3 expression. As shown in **Figure 7C and D**, we found that miR-16-5p reduced Dsg3 mRNA and protein expression levels in HaCaT cells. Thus, these results indicate that Dsg3 is a target gene of miR-16-5p.

To determine whether Dsg3 mediates the role of miR-16-5p in keratinocytes migration, the Dsg3 siRNA and Dsg3 overexpressing plasmid was respectively employed to regulate the expression of Dsg3. Cell migration was significantly promoted when Dsg3 expression was knocked down (**Figure 7E**). What's more, overexpression of Dsg3 restrained the cell migration that was promoted by miR-16-5p mimics (**Figure 7E**). Waschke *et al.* has reported that Dsg3 regulates wound repair in a p38/MAPK-dependent manner 34. Next, we sought to investigate whether miR-16-5p affected migration *via* the p38 signaling pathway. As shown in **Figure 7F**, we found that the relative expression of p-p38/p38 was significantly increased in the miR-16-5p mimics or Dsg3 siRNA treated group compared with the control group, while overexpression of Dsg3 restrained the p38 activation that was promoted by miR-16-5p mimics. To further determine the role of p38 signaling in the miR-16-5p promoting keratinocyte migration, the activation of p38/MAPK was blocked using the p38/MAPK-specific inhibitor SB202190 at 30 μM. As evidenced by scratch wound assay, acceleration of gap closure in miR-16-5p mimics treated group was prevented by SB202190 (**Figure 7G**). Taken together, these data suggest that miR-16-5p promotes keratinocytes migration by targeting Dsg3, in which p38/ MAPK signaling pathway is involved.

### Acceleration of deep second-degree burn wound healing by miR-16-5p

**Figure 8:**
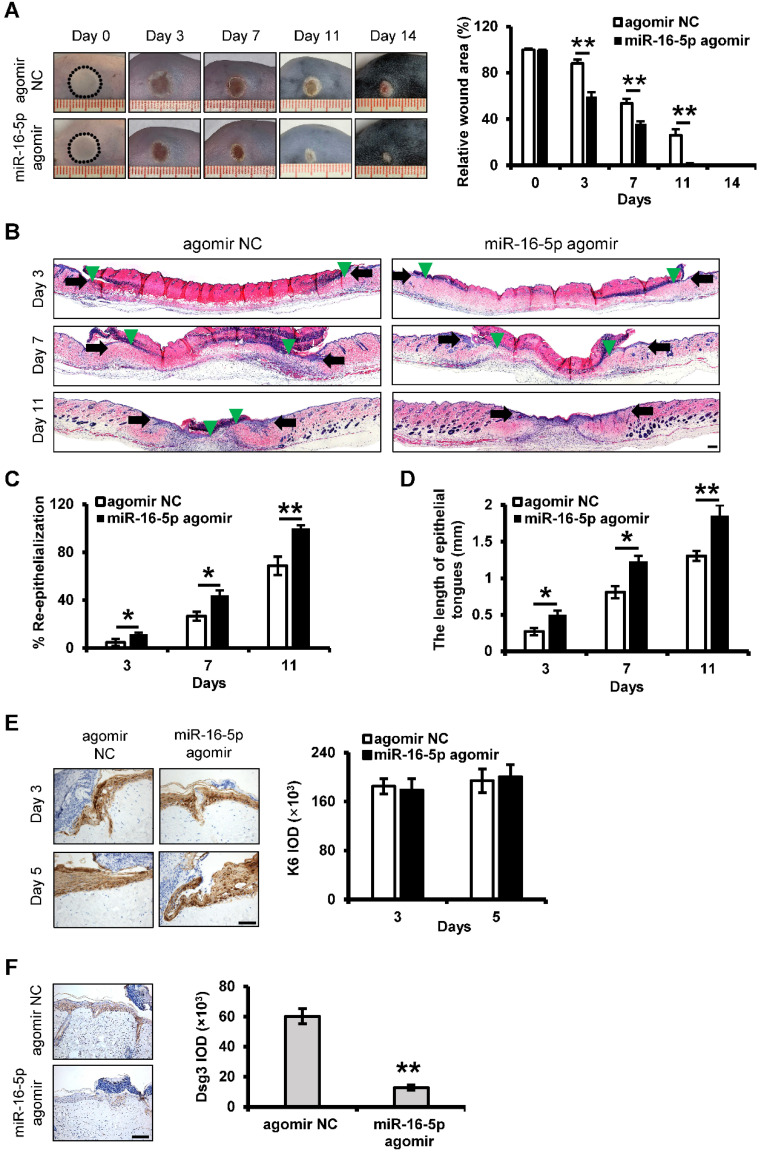
***In vivo* delivery of miR-16-5p accelerates deep second-degree burn wound healing in mice.** (**A**) Representative macroscopic images of wounds treated with miRNA agomir negative control (agomir NC) or miR-16-5p agomir on days 0, 3, 7, 11 and 14 after wounding. (left panel). Quantitative analysis of wound area per group, expressed as the percentage of the initial wound size at day 0 (right panel). n = 6 mice per group. (**B**) Representative photomicrographs of H&E-stained wounds treated with agomir NC or miR-16-5p agomir on days 3, 7 and 11 after wounding. Black arrows represent the dermal border; green arrows represent the epidermal margin. Scale bar = 200 µm. (**C**) Quantitative profiles of the re-epithelialization ration of wounds per group. (**D**) Quantitative profiles of the length of epithelial tongues of wounds per group. (**E**) Representative photomicrographs of immunohistochemical staining for K6 of wounds treated with agomir NC or miR-16-5p agomir on day 3 and 5 after wounding (left panel). Scale bars = 50 µm. The areas stained with K6 were determined by planimetric image analysis using Image Pro Plus 6.0 software. (**F**) Representative photomicrographs of immunohistochemical staining for Dsg3 of wounds treated with agomir NC or miR-16-5p agomir on days 5 after wounding (left panel). The areas stained with Dsg3 were determined by planimetric image analysis using Image Pro Plus 6.0 software (right panel). Scale bar = 100 µm. All values are expressed as mean ± SD from three independently repeats, *P < 0.05, **P < 0.01.

We next examined whether miR-16-5p treatment could exert beneficial effects on the burn wound healing. In this study, miR-16-5p agomir was used to overexpression of miR-16-5p in the burn wound site. Mice treated with miR-16-5p agomir showed greater wound closure than observed in the control groups at days 3, 7 and 11 post-wounding (**Figure 8A**). Furthermore, H&E staining of the wound site indicated significant increases in the degree of re-epithelialization of the wound and the length of epithelial tongues after 3, 7 and 11 days in the miR-16-5p agomir group compared to the control group (**Figure 8B-D**). Consistent with the effect of miR-16-5p on keratinocytes proliferation *in vitro*, we found that miR-16-5p did not affect keratinocytes proliferation at wound edge by K6 immunostaining (**Figure 8E**), suggesting that the enhanced re-epithelialization caused by miR-16-5p was possibly due to the promoted keratinocytes migration. In addition, we found expression levels of Dsg3 were decreased at wound edge treated with miR-16-5p compared to the control wound edge (**Figure 8F**), indicating that miR-16-5p promoted keratinocytes migration possibly *via* inhibiting the expression of Dsg3 in re-epithelialization process. Similar results were found in wound tissues treated with iPSCs-MVs compared with the PBS group (**Figure S1**).

We also examined the impact of miR-16-5p on other biological processes in wound healing. The results showed that there were no apparent differences in expressions of CD68 and α-SMA and the numbers of newly formed vessels between miR-16-5p agomir group and control group (**Figure S2A-C**). However, less collagen deposition was observed in the wounds treated with miR-16-5p agomir on days 7 and 11 (**Figure S2D**).

## Discussion

In this study, we demonstrated that iPSCs-MVs could accelerate deep second-degree burn wound healing in mice by affecting the migration of keratinocytes. We further showed that miR-16-5p, the miRNA present in the highest representation, was responsible for a large part, but possible not all, of the pro-migratory effect of iPSCs-MVs partially *via* directly targeting Dsg3. In addition, local treatment of miR-16-5p could accelerate wound close which was associated with increased re-epithelialization.

We successfully isolated iPSCs-MVs, confirmed by their diameter and smooth spherical shape structure. Our results showed that iPSCs-MVs significantly reduced the wound size and accelerated wound healing *in vivo*. Cutaneous wound healing in adult mammals is a complex multi-step process involving overlapping stages of blood clot formation, inflammation, re-epithelialization, granulation tissue formation, neovascularization, and remodeling. In our study, it was clearly observed that the application of iPSCs-MVs greatly increased re-epithelization, myofibroblasts and collagen deposition at wound sites. Meanwhile, iPSCs-MVs promoted the generation of newly formed vessels. These results suggested that iPSCs-MVs would be a superior candidate for treating burn wound healing that might overcome the obstacles and risks associated with stem cell transplantation therapy.

Re-epithelization is the resurfacing of a wound with new epithelium and consists of both migration and proliferation of keratinocytes at the periphery of the wound 35. As epidermal migration moves on, keratinocytes at the wound margin begin to proliferate behind the actively migrating cells 36. The results of our study showed that the effect of iPSCs-MVs on wound closure was due to enhanced keratinocytes migration but not proliferation during the healing process. We further evaluated the effects of iPSCs-MVs on the behavior of keratinocytes *in vitro*. The results revealed that these nanoparticles could be internalized by keratinocytes and significantly promote their migration without affecting cell proliferation. Thus, the beneficial effects of iPSCs-MVs on wound healing may be mainly attributed to their function on promotion of keratinocytes migration.

It has been shown that extracellular vesicles contain large amounts of miRNAs and can serve as vehicles to transfer miRNAs to recipient cells, where the exogenous miRNAs can alter the gene expression and bioactivity of recipient cells. In our data, using high-throughput sequencing and functional analysis, we detected several highly abundant specific microRNAs derived from iPSCs-MVs. Among them, similar to iPSCs-MVs, miR-16-5p has been shown to significantly promote migration of HaCaT cells. Furthermore, after incubating with iPSCs-MVs, we found that miR-16-5p expression was remarkable enhanced in keratinocytes. In addition, the iPSCs-MVs-induced promotion of keratinocytes migration was attenuated by miR-16-5p inhibitor. Thus, we believe that miR-16-5p is one of the critical mediators in iPSCs-MVs-induced regulation of keratinocytes migration.

Previously, many studies have reported that microRNAs play very important roles in the proliferation and migration of keratinocytes such as miR-21, miR-198 and miR-210 37-39. However, due to the large number of miRNAs expressed in keratinocytes, our understanding of miRNAs in keratinocytes regulation is not enough. Evidence in this study demonstrates the important role of miR-16-5p in keratinocytes. miR-16 has been reported to play a different role in the functions of different types of tumor cells by regulating a variety of mRNA target genes. For example, miR-16 was downregulated in osteosarcoma 40, lung cancer 39, chronic lymphocytic lymphoma 40, and gastric cancer 43 and inhibited proliferation, invasion and migration in many types of cancer cells. However, some reports showed the opposite views. Zhu *et al.* found that miR-16 induced the suppression of cell apoptosis while promote proliferation in esophageal squamous cell carcinoma 44. The study from Wu *et al.* demonstrated that miR-16 targeted Zyxin and promoted cell motility in human laryngeal carcinoma cell line HEp-2 45. The role of miR-16-5p in keratinocytes has not been reported. Interestingly, evidence in this study demonstrated that miR-16-5p enhanced keratinocytes migration but had no effect on keratinocytes proliferation, suggesting that the effect of miR-16 might be diverse among different cells from different organs and tissues.

We report that Dsg3, as a new target gene of miR-16-5p, is involved in miR-16-5p mediated promotion of migration in HaCaT cells. Dsg3 is one of seven desmosomal cadherins, of two subfamilies, which have been identified in human tissues comprising four desmogleins (Dsg1-4) and three desmocollins (Dsc1-3) 46. These desmosomes in keratinocytes are the most important intercellular adhering junctions that provide structural strength for the epidermis. Rötzer* et al.* had shown that human keratinocytes after silencing of Dsg3 as well as primary cells isolated from Dsg3 knockout animals exhibited accelerated migration, which was further corroborated in an *ex vivo* skin outgrowth assay. In addition, their data suggested that Dsg3 controls a switch from an adhesive to a migratory keratinocytes phenotype *via* p38/MAPK inhibition 34. Consistent with these results, our observations confirmed that keratinocytes migration could be promoted by Dsg3 knockdown. In this study, RT-PCR, Western blot and Luciferase reporter activity assay all demonstrated that Dsg3 was a direct target gene of miR-16-5p in HaCaT cells. In addition, rescue experiment revealed that overexpression of Dsg3 reversed the cell migration that was promoted by miR-16-5p. Furthermore, the activations of p38/MAPK pathways were found to be significantly induced by miR-16-5p overexpression, which further demonstrated that Dsg3 was a target of miR-16-5p in HaCaT cells. This is the first time that miR-16-5p has been shown to promote keratinocytes migration. However, our study has limitations because miRNAs have multiple target genes. This fact does not exclude miR-16-5p from regulating keratinocytes migration through other target genes. We have confirmed only that Dsg3 plays a role in this process, but the other underlying mechanism is unclear.

Based on *in vitro* results, we applied local treatment of miR-16-5p in the burn wound site. We found that miR-16-5p significantly shrieked the wound size, accelerated wound healing, and promoted re-epithelialization *in vivo*. Supportively, Dsg3, direct target of miR-16-5p, exhibited the inverse correlation with miR-16-5p expression at wound edge during healing process, indicating that miR-16-5p promoted keratinocytes migration possibly* via* inhibiting the expression of Dsg3 in re-epithelialization process.

In addition to promoting reepithelization, we found that the effects of miR-16-5p on burn healing were not completely consistent with iPSCs-MVs. In the wounds treated with miR-16-5p, inflammation and angiogenesis were not affected, while collagen deposition was reduced. Interesting, several studies reported that miR-16-5p could suppress TGF-β signaling pathway 47-48, which is closely associated with collagen deposition and hypertrophic scar formation. Thus, further studies are required to explore the possibility of miR-16-5p affecting scar formation during wound healing.

In summary, our findings indicate that both iPSCs-MVs and iPSCs-MVs-derived miR-16-5p could be effective on wound re-epithelialization, which would hold great potential for the treatment of wounds, especially chronic wounds. Further, we will continue to explore the possibility of iPSCs-MVs affecting other different types of cells in skin wound.

## Supplementary Material

Supplementary figures and tables.

## Abbreviations

iPSCs: induced pluripotent stem cells
MVs: microvesicles
iPSCs-MVs: iPSCs-derived microvesicles
MSCs: mesenchymal stem cell
miRNAs: microRNAs
ESCs: epidermal stem cells
DMEM: Dulbecco's modified Eagle's medium
PBS: phosphate-buffered saline
TEM: transmission electron microscopy
NTA: nanoparticle tracking analysis
DAPI: 4,6-diamidino-2-phenylindole
EdU: 5-Ethynyl-2'-deoxyuridine
SDS-PAGE: sodium dodecyl sulfate-polyacrylamide gel electrophoresis
qRT-PCR: quantitative real-time polymerase chain reaction
H&E: hematoxylin and eosin
K6: cytokeratin-6
Dsg3: desmoglein 3
MAPK: mitogen activated protein kinases
