# Supplementary material for: Induced pluripotent stem cells-derived microvesicles accelerate deep second-degree burn wound healing in mice through miR-16-5p-mediated promotion of keratinocytes migration
Source: Theranostics. 2020 Aug 8;10(22):9970–83. doi: 10.7150/thno.46639 (PMC7481429; doi:10.7150/thno.46639)
Supplement: Supplementary file 1 — Supplementary figures and tables. [file thnov10p9970s1.pdf]

Table S1

Highly expressed miRNAs in iPSCs-MVs.

Gene ID	Name	Read count
MIMAT0000527	mmu-miR-16-5p	603437
MIMAT0000540	mmu-miR-93-5p	197446
MIMAT0000212	mmu-miR-183-5p	133357
MIMAT0000542	mmu-miR-34a-5p	122625
MIMAT0000532	mmu-miR-23a-3p	120342
MIMAT0000513	mmu-miR-19b-3p	99921
MIMAT0000126	mmu-miR-27b-3p	77640
MIMAT0000677	mmu-miR-7a-5p	76904
MIMAT0000534	mmu-miR-26b-5p	71735
MIMAT0000652	mmu-miR-25-3p	71284
MIMAT0000122	mmu-let-7i-5p	57868
MIMAT0000379	mmu-miR-301a-3p	46453
MIMAT0000537	mmu-miR-27a-3p	45089
MIMAT0000133	mmu-miR-101a-3p	42264
MIMAT0000158	mmu-miR-146a-5p	41837
MIMAT0000546	mmu-miR-103-3p	40494
MIMAT0000533	mmu-miR-26a-5p	40464
MIMAT0000387	mmu-miR-130b-3p	38881
MIMAT0000539	mmu-miR-92a-3p	32863
MIMAT0000141	mmu-miR-130a-3p	31745
MIMAT0000530	mmu-miR-21a-5p	28956
MIMAT0000208	mmu-miR-10b-5p	28812
MIMAT0000215	mmu-miR-186-5p	26603
MIMAT0000219	mmu-miR-24-3p	23834
MIMAT0000221	mmu-miR-191-5p	23551
MIMAT0001081	mmu-miR-196b-5p	23013
MIMAT0000516	mmu-miR-148a-3p	22984
MIMAT0000651	mmu-miR-19a-3p	20627
MIMAT0000541	mmu-miR-96-5p	18947
MIMAT0004186	mmu-miR-301b-3p	14963

Figure S1 iPSCs-MVs decreased the expression of Dsg3 during burn wound healing. (**A**) Representative photomicrographs of immunohistochemical staining for Dsg3 of wounds treated with PBS or iPSCs-MVs on days 5 after wounding. Scale bar = 50 µm. (**B**) The areas stained with Dsg3 were determined by planimetric image analysis using Image Pro Plus 6.0 software. All values are expressed as mean ± SD from three independently repeats , **P < 0.01.

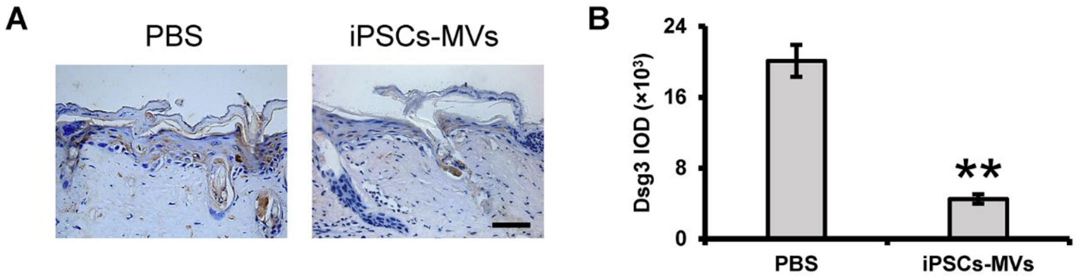

Figure S2 Effects of mir-16-5p on inflammation, fibrosis and angiogenesis during burn wound healing. (**A**) Representative photomicrographs of immunohistochemical staining for CD68 of wounds treated with agomir NC or miR-16-5p agomir on days 3 and 5 after wounding (left panel). Scale bar = 50 μm. The areas stained with CD68 were determined by planimetric image analysis using Image Pro Plus 6.0 software (right panel). (**B**) Representative photomicrographs of immunohistochemical staining for CD31 of wounds treated with agomir NC or miR-16-5p agomir on days 11 after wounding (left panel). Scale bar = 50 μm. The number of stained capillaries were counted (right panel). Statistics regarding the number of stained capillaries were obtained using five randomly selected fields of view for each group. (**C**) Representative photomicrographs of immunohistochemical staining for α-SMA of wounds treated with agomir NC or miR-16-5p agomir on days 5 and 9 after wounding (left panel). Scale bar = 50 μm. The areas stained with α-SMA were determined by planimetric image analysis using Image Pro Plus 6.0 software (right panel). (**D**) Representative photomicrographs of Masson's trichrome -stained wounds treated with agomir NC or miR-16-5p agomir on days 7 and 11 after wounding. All values are expressed as mean ± SD from three independently repeats.

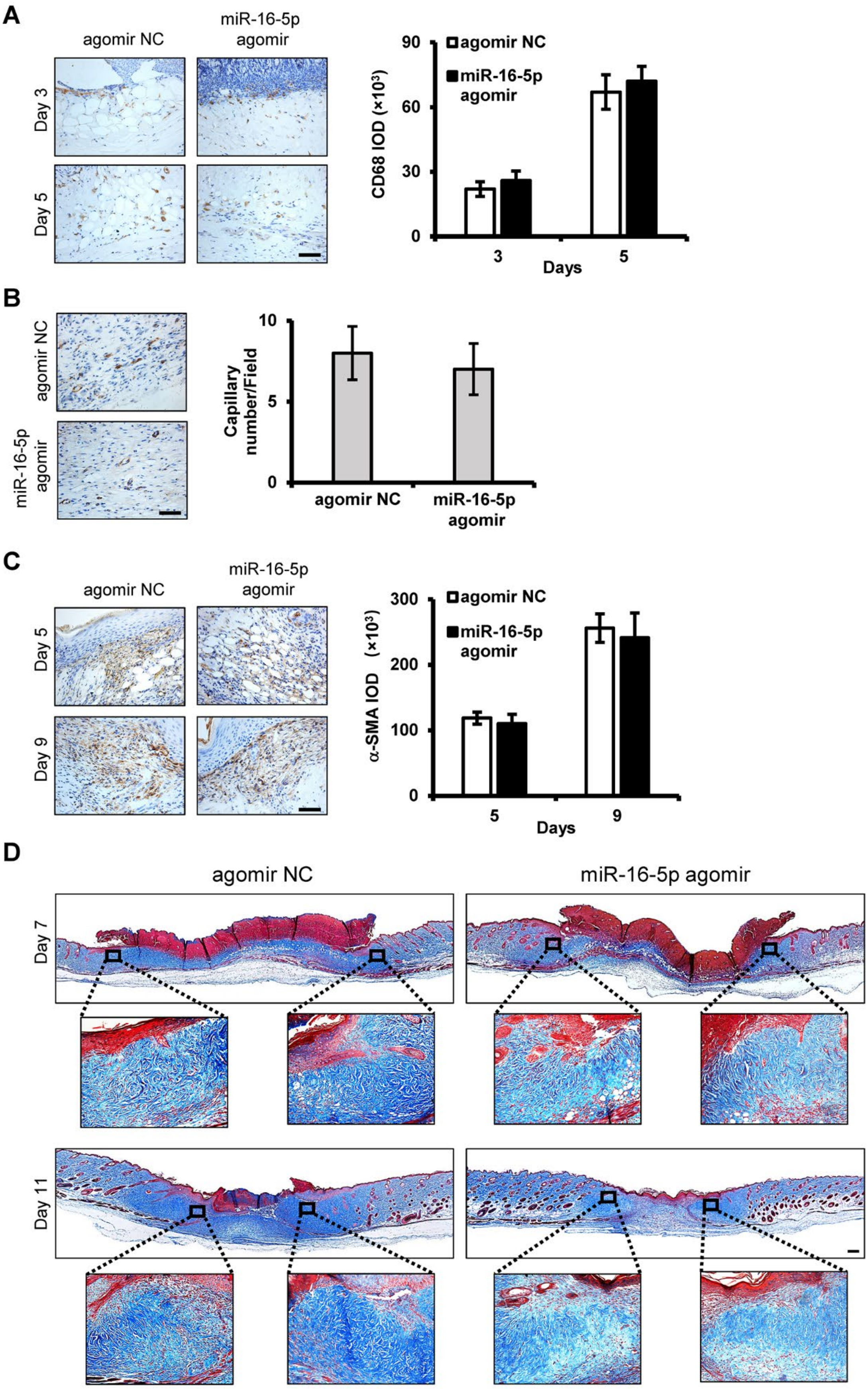

A
agomir NC
miR-16-5p agomir
Day 3
Day 5
CD68 IOD (×10³)
agomir NC
miR-16-5p agomir
90
60
30
0
3
5
Days
B
agomir NC
miR-16-5p agomir
Capillary number/Field
10
5
0
C
agomir NC
miR-16-5p agomir
Day 5
Day 9
α-SMA IOD (×10³)
300
200
100
0
5
9
Days
D
agomir NC
miR-16-5p agomir
Day 7
Day 11
